# Effects of right ventricular remodeling in chronic thromboembolic pulmonary hypertension on the outcomes of balloon pulmonary angioplasty: a 2D-speckle tracking echocardiography study

**DOI:** 10.1186/s12931-024-02803-4

**Published:** 2024-04-15

**Authors:** Yaning Ma, Dichen Guo, Jianfeng Wang, Juanni Gong, Huimin Hu, Xinyuan Zhang, Yeqing Wang, Yuanhua Yang, Xiuzhang Lv, Yidan Li

**Affiliations:** 1 Department of Ultrasound Medicine, Beijing Chao-Yang Hospital, Capital Medical University, 8 Gongren Tiyuchang Nanlu, Chaoyang District, Beijing, 100020 China; 2 Department of Intervention, Beijing Chao-Yang Hospital, Capital Medical University, Beijing, China; 3 Department of Respiratory and Critical Care Medicine, Beijing Chao-Yang Hospital, Capital Medical University, Beijing, China

**Keywords:** Chronic thromboembolic pulmonary hypertension, Balloon pulmonary angioplasty, Right ventricular remodeling, Right ventricular free wall longitudinal strain, Predictor

## Abstract

**Background:**

Balloon pulmonary angioplasty (BPA) improves the prognosis of chronic thromboembolic pulmonary hypertension (CTEPH). Right ventricle (RV) is an important predictor of prognosis in CTEPH patients. 2D-speckle tracking echocardiography (2D-STE) can evaluate RV function. This study aimed to evaluate the effectiveness of BPA in CTEPH patients and to assess the value of 2D-STE in predicting outcomes of BPA.

**Methods:**

A total of 76 patients with CTEPH underwent 354 BPA sessions from January 2017 to October 2022. Responders were defined as those with mean pulmonary artery pressure (mPAP) ≤ 30 mmHg or those showing ≥ 30% decrease in pulmonary vascular resistance (PVR) after the last BPA session, compared to baseline. Logistic regression analysis was performed to identify predictors of BPA efficacy.

**Results:**

BPA resulted in a significant decrease in mPAP (from 50.8 ± 10.4 mmHg to 35.5 ± 11.9 mmHg, *p* < 0.001), PVR (from 888.7 ± 363.5 dyn·s·cm^−5^ to 545.5 ± 383.8 dyn·s·cm^−5^, *p* < 0.001), and eccentricity index (from 1.3 to 1.1, *p* < 0.001), and a significant increase in RV free wall longitudinal strain (RVFWLS: from 15.7% to 21.0%, *p* < 0.001). Significant improvement was also observed in the 6-min walking distance (from 385.5 m to 454.5 m, *p* < 0.001). After adjusting for confounders, multivariate analysis showed that RVFWLS was the only independent predictor of BPA efficacy. The optimal RVFWLS cutoff value for predicting BPA responders was 12%.

**Conclusions:**

BPA was found to reduce pulmonary artery pressure, reverse RV remodeling, and improve exercise capacity. RVFWLS obtained by 2D-STE was an independent predictor of BPA outcomes. Our study may provide a meaningful reference for interventional therapy of CTEPH.

## Introduction

Chronic thromboembolic pulmonary hypertension (CTEPH) is a severe disease characterized by obstructive pulmonary artery remodeling due to insoluble embolus or repeated embolization of a thrombus in proximal or distal arteries [1]. Long-term afterload in these patients induces right ventricular (RV) maladaptive remodeling including eccentric hypertrophy and myocardial dysfunction, leading to right heart failure and even death [2, 3]. The reported annual incidence and prevalence of CTEPH are 2–6 and 26–38 cases/million adults, respectively [4]. The treatment modalities for CTEPH include medical therapy, balloon pulmonary angioplasty (BPA), and pulmonary endarterectomy (PEA). Although the results of BPA were shown to be inferior to those of PEA, BPA is currently the preferred treatment for inoperable CTEPH and recurrent PH after PEA [5]. BPA can improve RV function, hemodynamics, and exercise capacity by mechanically dilating the narrowed or occluded pulmonary artery. Despite the promising outcomes of BPA, evidence is still scarce.

RV fibrosis plays an important role in RV adaptive and maladaptive remodeling [6, 7]. This process impairs RV contraction and relaxation and is associated with disease severity in patients with CTEPH [8]. Cardiopulmonary hemodynamics assessed by right cardiac catheterization (RHC) is an important predictor of prognosis [1]. However, RHC is not routinely performed due to its invasive nature and high cost. Several other factors affect the outcomes of BPA in CTEPH patients, of which RV dysfunction is a major determinant [9, 10]. Two-dimensional speckle tracking echocardiography (2D-STE) is a sensitive imaging modality for assessing RV function [11]. Echocardiography is a widely accessible and low-cost investigation for the evaluation of right-sided heart in CTEPH. Therefore, there is a need for parameters that can predict outcomes.

There were two main objectives of this study. The first objective was to evaluate the effect of BPA on RV reverse remodeling. The second objective was to analyze the differences in baseline characteristics between BPA responders and non-responders and to identify factors that contribute to this difference. The overarching aim of this study was to identify patients who are more likely to respond to BPA.

## Materials and methods

### Study design and population

This was a single-center retrospective study. The study protocol was approved by the Ethics Committee of Beijing Chaoyang Hospital. All patients were informed in detail about the study and their written informed consent was obtained.

**Fig. 1:**
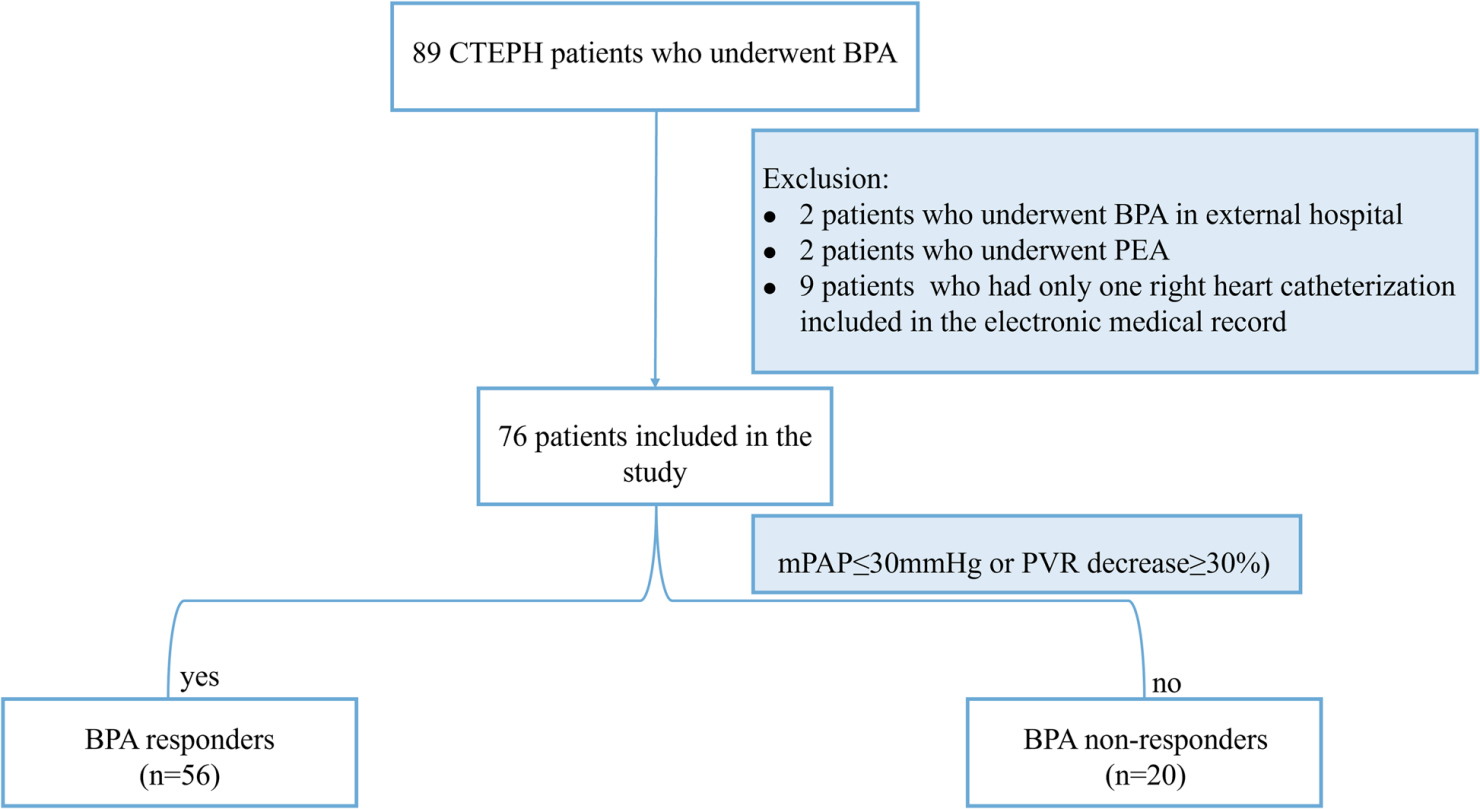
Study population selection. BPA, Balloon pulmonary angioplasty

We screened CTEPH patients who underwent BPA from January 2017 to October 2022 at the Beijing Chaoyang Hospital. The diagnosis of CTEPH was according to the 2022 European Society of Cardiology/European Respiratory Society (ESC/ERS) guidelines for pulmonary hypertension (PH). A total of 89 CTEPH patients were eligible for BPA treatment during the study period. Out of these, 13 patients were excluded because of the following reasons: BPA was performed at another hospital (*n* = 2); patients treated with PEA (*n* = 2); records of only one RHC were available (*n* = 9). Therefore, 76 patients with CTEPH were enrolled in the study (Fig. 1). All patients underwent RHC and echocardiography before their first BPA session (baseline) and within 3–6 months after their last BPA session. Clinical data were collected by two independent reviewers from the electronic medical record system. Based on the published literature, patients with mPAP ≤ 30 mmHg or those showing a PVR decrease of ≥ 30% compared to baseline were defined as responders, while the others were defined as non-responders [12].

### Echocardiographic study of the right heart

An experienced echocardiologist performed all conventional 2D and Doppler examinations using a commercially available ultrasound system (Philips EPIQ 7C, Philips Healthcare, MA, USA) equipped with X5-1 phased array transducers. The echocardiologist was blinded to patient characteristics. Five consecutive cardiac cycles in sinus rhythm were recorded using lead III echocardiogram. The examination was performed according to the American Society of Echocardiography (ASE) guidelines and 2022ESC/ERS guidelines for PH [4, 13]. Conventional parameters of RV structure and function were recorded. Structural parameters include the left ventricular eccentricity index (LVEI), RV end-diastolic area (RVEDA), RV end-systolic area (RVESA), and RV basal diameter; the functional parameters include tricuspid annular plane systolic excursion (TAPSE), peak systolic velocity of the tricuspid annulus (S'), RV index of myocardial performance (RIMP), and RV fractional area change (FAC). Data regarding pulmonary artery systolic pressure (PASP) and the diameters of the main pulmonary artery and its branches were also collected.

### 2D speckle tracking echocardiography

**Fig. 2:**
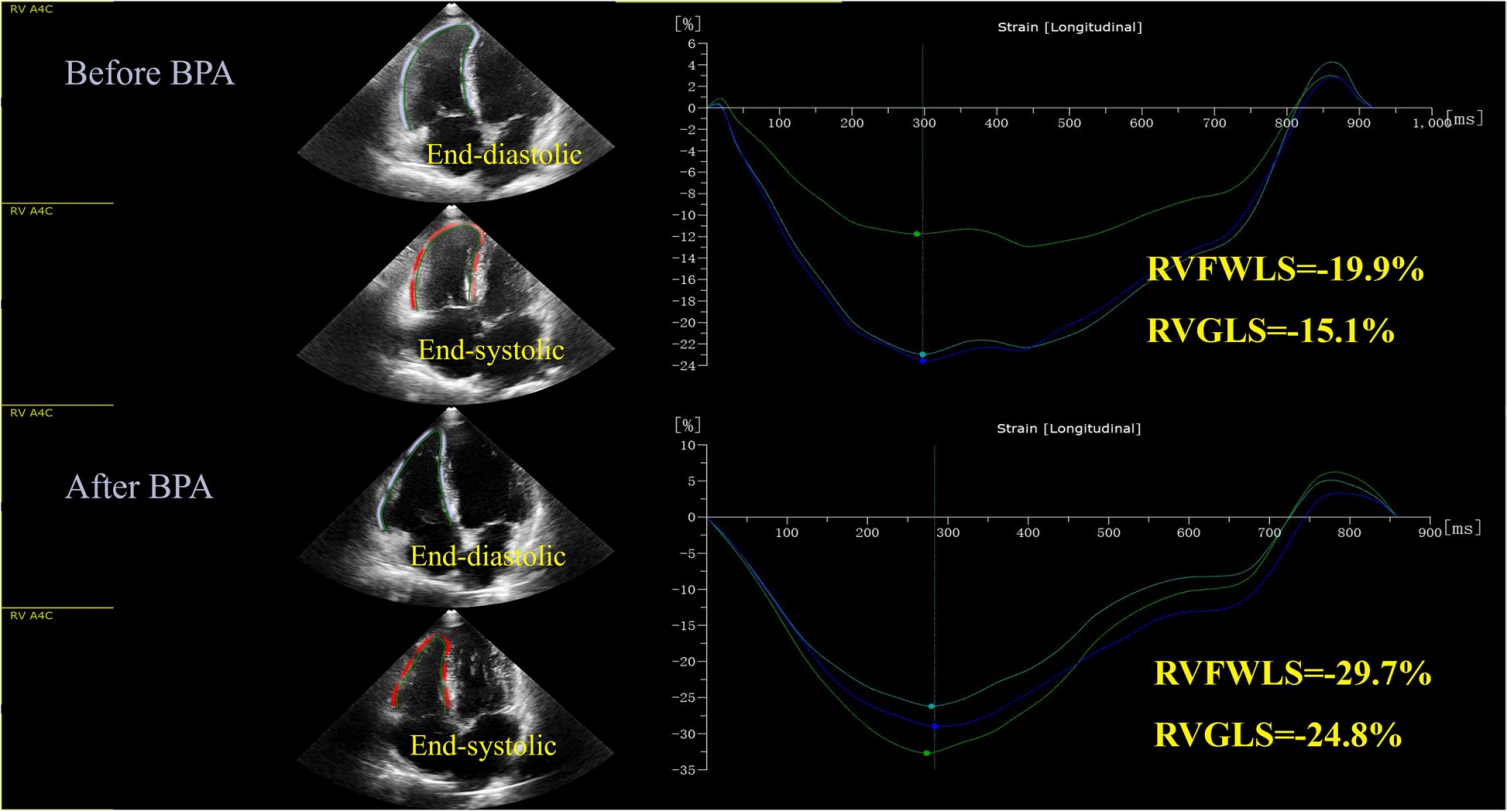
Right ventricular longitudinal strain measured by 2D-speckle tracking echocardiography (2D-STE) before and after balloon pulmonary angioplasty (BPA) in a patient. RVFWLS, RV free wall longitudinal strain; RVGLS, RV global longitudinal strain

RV strain was analyzed offline using QLAB 15.0 (Philips, Andover, MA) by an experienced physician in the RV focus four-chamber view. The software automatically tracks a region of interest, and if it fails, the region of interest can be manually marked by tracing the RV endocardial boundary. RV strain analysis was performed by tracing from the tricuspid annulus to the RV apex. The software automatically calculates the RV free wall longitudinal strain (RVFWLS) and RV global longitudinal strain (RVGLS). All strain values were expressed as absolute values. An example of RV strain measurement using 2D-STE in a CTEPH patient was presented in Fig. 2.

### Right heart catheterization

The RHC is the gold standard for evaluating pulmonary hemodynamics and is necessary for the diagnosis of PH [14]. RHC is an interventional technique involving the insertion of a cardiac catheter into the right heart system via peripheral veins. RHC was performed using Swan–Ganz catheter via the internal jugular vein. The measured hemodynamic parameters included central venous pressure (CVP), mean pulmonary artery pressure (mPAP), pulmonary vascular resistance (PVR), pulmonary capillary wedge pressure (PCWP), cardiac output (CO), and cardiac index (CI).

### Balloon pulmonary angioplasty

**Fig. 3:**
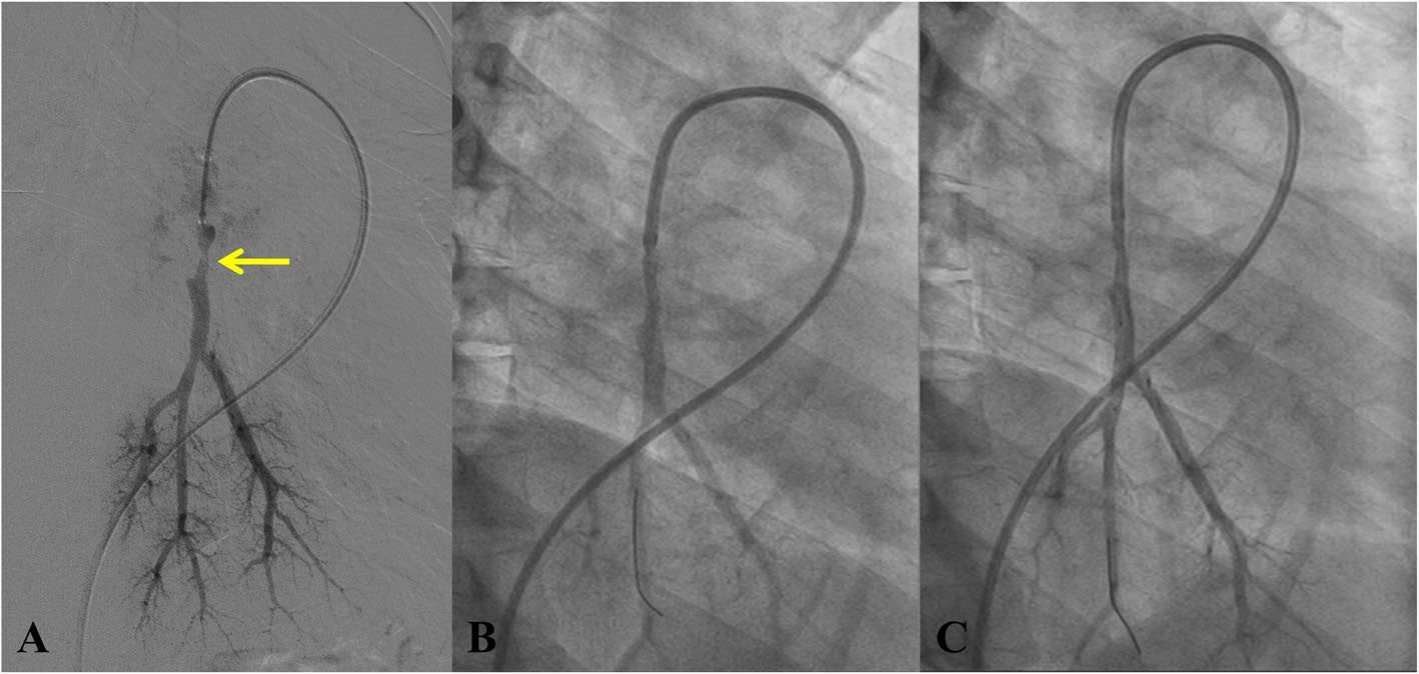
Pulmonary angiography before and after BPA in a patient. **A** Pulmonary angiography before BPA. The yellow arrow indicateed the pulmonary artery stenosis; **B** Pulmonary angiography after 2.5 mm balloon dilation; **C** Pulmonary angiography after 4 mm balloon dilation

The BPA is a staged procedure to dilate a limited number of blocked segmental or subsegmental pulmonary arteries [15]. BPA has been shown to be a safe and effective treatment for inoperable CTEPH [16]. After applying a local anesthetic, an 8F lower-extremity arterial sheath was inserted into the pulmonary artery through the femoral or jugular access. The guidewire was delivered through the catheter to the targeted segmental arteries, and the guidewire was retained through the catheter. A suitable size balloon was selected and navigated to the targeted arteries via the guidewire. The balloon was inflated to dilate the narrowed or obstructed pulmonary arteries. The therapeutic effect was assessed by pulmonary angiography, and postoperative residual stenosis < 30% was considered a sign of treatment success. The total number of BPA procedures was tailored based on the overall assessment of the doctor and patient’s response to BPA. An example of pulmonary angiography in a CTEPH patient was presented in Fig. 3.

### Statistical analysis

Continuous variables are presented as mean ± standard deviation (SD) or as median and interquartile range, based on the data distribution. Categorical data are expressed as frequency and percentage. Student’s *t*-test or Mann–Whitney U test was applied for analyzing inter-group differences regarding continuous variables. Fisher’s exact test or Chi-square test was used for categorical variables. All potential covariates of interest were included in a univariable logistic regression model. Variables that showed a significant association (*p* < 0.05) in the univariable logistic regression were included in the multivariable logistic regression model. Receiver operating characteristic (ROC) curve analysis was performed to assess the accuracy of the model and calculate the optimal cutoff value. Bland–Altman analysis was used to analyze intra-observer and inter-observer reproducibility. Statistical analysis was performed using SPSS version 25 (SPSS Inc., Chicago, IL, USA) and GraphPad Prism 8 (GraphPad, La Jolla, CA). Two-sided *p* values < 0.05 were considered indicative of statistical significance.

## Results

### Population cohort

The baseline characteristics are summarized in Table 1. A total of 354 BPA sessions [5(3)/per patient] were performed in our cohort. The median time interval of BPA was 331 days. The median age of patients was 60 years and 67% were female. Left heart function was normal in all patients (mean LVEF 65.2 ± 5.1%). According to hemodynamic evaluation after the final BPA session, 56 patients were categorized as BPA responders and 20 patients as non-responders.

**Table 1:** Baseline characteristics of all patients, BPA responders and non-responders

Variables	All patients (*n* = 76)	Responders (*n* = 56)	Non-responders (*n* = 20)	*P*-value
Demographics				
Age, years	59.5 (16.0)	58.2 ± 10.9	55.2 ± 14.4	0.341
Female, n (%)	51 (67.1)	38 (67.9)	13 (65.0)	0.815
BSA, cm^2^	1.7 (0.2)	1.7 ± 0.1	1.7 (0.3)	0.706
Echocardiography				
PASP, mmHg	86.9 ± 20.5	84.3 ± 21.1	94.1 ± 17.0	0.066
TAPSE, mm	14.8 ± 3.3	15.3 ± 3.3	13.4 ± 2.8	0.030
LVEI	1.3 (0.2)	1.3 (0.2)	1.5 ± 0.2	0.029
TRV, cm/s	433 ± 58.1	425.9 ± 59.9	456.0 ± 47.2	0.046
D_IVC_, mm	22.4 ± 4.7	22.0 ± 4.8	23.7 ± 4.1	0.164
S', cm/s	9.1 (2.5)	9.1 (3.0)	9.1 (1.5)	0.729
RIMP	0.8 (0.3)	0.7 (0.3)	0.8 (0.2)	0.051
FAC, %	29.4 ± 9.9	30.8 ± 9.9	25.7 ± 9.2	0.050
RVEDA, cm^2^	25.3 ± 7.2	23.9 ± 6.9	29.1 ± 6.9	0.006
RVESA, cm^2^	18.2 ± 6.8	16.9 ± 6.4	21.8 ± 6.6	0.006
RV basal diameter, mm	45.9 ± 6.9	45.0 ± 6.7	47.5 (7.5)	0.119
D_MPA_, mm	32.0 (6.2)	31.0 (6.2)	33.2 (7.0)	0.296
D_RPA_, mm	24.0 (4.4)	23.0 (4.5)	24.9 ± 3.8	0.174
D_LPA,_ mm	22.0 (4.0)	21.6 (3.4)	22.9 ± 3.8	0.225
LVEF, %	65.2 ± 5.1	65.0 ± 5.5	66.0 ± 3.9	0.456
Strain parameters				
STE-RVFWLS, %	15.7 (7.6)	16.7 (6.1)	10.0 (2.3)	< 0.001
STE-RVGLS, %	13.4 ± 4.4	14.1 (5.6)	8.7 (2.6)	< 0.001
Hemodynamics				
CVP, mmHg	8.1 ± 4.1	6.5 (5.0)	9.0 ± 3.2	0.154
mPAP, mmHg	50.8 ± 10.4	50.3 ± 10.5	52.1 ± 10.3	0.547
PVR, dyn·s·cm^−5^	888.7 ± 363.5	879.6 ± 373.2	914.4 ± 342.8	0.528
PCWP, mmHg	9.0 (5.5)	9.0 (6.0)	10.0 (8.0)	0.426
CO, L/min	3.9 (1.4)	4.1 ± 1.1	3.9 ± 1.3	0.640
CI, L/min/m^2^	2.3 ± 0.5	2.3 ± 0.5	2.2 ± 0.5	0.564
Clinical indicators				
WHO FC				
I/II/III/IV	4/40/29/3	2/35/16/1	2/5/13/2	0.001
6WMD, m	385.5 (126.0)	393.0 (104.0)	312.0 (232.5)	0.019
NT-proBNP, ng/L	675.0 (1858.0)	542 (1214.0)	1647 (2441.8)	0.011
BPA procedures				
Number of BPA sessions	5 (3)	5 ± 2	4 ± 2	0.465
Time Interval, days	331.0 (446.0)	331.0 (472.3)	334.5 (439.0)	0.986

Values are expressed as mean ± SD, median (IQR), or frequency (percentage), as appropriate

*Abbreviations*: *BSA* Body surface area, *PASP* Pulmonary artery systolic pressure by echocardiography, *TAPSE* Tricuspid annular plane systolic excursion, *LVEI* Left ventricular eccentric index, *TRV* Tricuspid regurgitation peak velocity, *D*_*IVC*_ Diameter of inferior vena cava; S', peak systolic velocity of the tricuspid annulus, *RIMP* Right ventricular index of myocardial performance, *FAC* Right ventricular fractional area change, *RVEDA* RV end-diastolic area, *RVESA* RV end-systolic area, *D*_*MPA*_ Main pulmonary artery diameter, *D*_*RPA*_ Right pulmonary artery diameter, *D*_*LPA*_ Left pulmonary artery diameter, *LVEF* Left ventricle ejection fraction, *STE-RVFWLS* Speckle tracking echocardiography-RV free wall longitudinal strain, *STE-RVGLS* Speckle tracking echocardiography-RV global longitudinal strain, *CVP* Central venous pressure; mPAP, mean pulmonary artery pressure, *PVR* Pulmonary vascular resistance, *PCWP* Pulmonary capillary wedge pressure, *CO* Cardiac output, *CI* Cardiac index, *WHO FC* World Health Organization Functional Class, *6MWD* 6 min walking distance, *NT-proBNP* N-terminal probrain natriuretic peptide, *BPA* Balloon pulmonary angioplasty

### Baseline characteristics of BPA responders and non-responders

There were no significant differences between responders and non-responders regarding the baseline demographics and BPA procedures. Baseline mPAP (50.3 ± 10.5 vs 52.1 ± 10.3 mmHg, *p* = 0.547) and PVR (879.6 ± 373.2 vs 914.4 ± 342.8 dyn·s·cm^−5^, *p* = 0.528) were also comparable between the two groups. However, the responders had better echocardiographic features (including TAPSE, EI, TRV, RVEDA, and RVESA) and clinical makers (including World Health Organization Functional classification [WHO FC], 6-m walking distance [6MWD], and N-terminal pro-brain natriuretic peptide [NT-proBNP] level) than non-responders. Besides, BPA responders had significantly higher levels of STE-RVFWLS (16.7% vs 10.0%, *p* < 0.001) and STE-RVGLS (14.1% vs 8.7%, *p* < 0.001) compared with BPA non-responders. 71 patients (93.4%) were treated with PH-targeted drugs and anticoagulants before BPA (Table 2). The remaining five patients were not treated with medication before BPA. Surprisingly, these five patients were all responders. Meanwhile, their baseline mPAP (47.8 mm Hg) and RVFWLS (19.3%) were better than general responders.

**Table 2:** PH drug therapy of all patients before BPA

PH drug therapy	N (%)
PH targeted therapy	
None	5 (6.58)
ERA	16 (2.67)
PDE-5 inhibitors	23 (30.26)
sGC stimulators	9 (11.84)
Prostacyclin analogues	8 (10.53)
CCB	2 (2.63)
Anticoagulation	
None	5 (6.58)
Warfarin	56 (73.68)
Rivaroxaban	20 (26.32)

Values are expressed as frequency (percentage)

*Abbreviations*: *PH* Pulmonary hypertension, *ERA* Endothelin receptor antagonists, *PDE-5 inhibitors* Phosphodiesterase-5 inhibitors, *sGC stimulators* Soluble guanylate cyclase stimulators, *CCB* Calcium channel blockers

### Comparison

A comparison of the pre- and post-BPA parameters is shown in Table 3. The mean mPAP showed a significant decrease after BPA (from 50.8 ± 10.4 mmHg to 35.5 ± 11.9 mmHg, *p* < 0.001). 16 patients (21%) achieved an mPAP ≤ 25 mmHg after BPA. The mean PVR decreased from 888.7 ± 363.5 dyn·s·cm^−5^ to 545.4 ± 383.8 dyn·s·cm^−5^ (*p* < 0.001), and the mean 6MWD significantly improved from 385.5 m to 454.5 m (*p* < 0.001). BPA resulted in a significant decrease in PASP (from 86.9 ± 20.5 mmHg to 56.6 mmHg, *p* < 0.001), EI (from 1.3 to 1.1, *p* < 0.001), and RIMP (from 0.8 to 0.6, *p* < 0.001), and a significant increase in TAPSE (from 14.8 ± 3.3 mm to 17.4 ± 3.5 mm), S' (from 9.1 cm/s to 10.6 cm/s, *p* < 0.001), FAC (from 29.4 ± 9.9% to 38.9 ± 10.0%, *p* < 0.001), RVFWLS (from 15.7% to 21.0%, *p* < 0.001), and RVGLS (from 13.4 ± 4.4% to 17.1 ± 5.1, *p* < 0.001).

**Table 3:** Comparison of pre-BPA and post-BPA parameters in all patients

Variables	Before BPA	After BPA	*P*-value
Echocardiography			
PASP, mmHg	86.9 ± 20.5	56.5 (32.6)	< 0.001
TAPSE, mm	14.8 ± 3.3	17.4 ± 3.5	< 0.001
LVEI	1.3 (0.2)	1.1 (0.2)	< 0.001
TRV, cm/s	433.0 ± 58.1	350.0 (109.0)	< 0.001
D_IVC_, mm	22.4 ± 4.7	20.3 (4.9)	0.008
S', cm/s	9.1 (2.5)	10.6 (3.3)	< 0.001
RIMP	0.8 (0.3)	0.6 (0.2)	< 0.001
FAC, %	29.4 ± 9.9	38.9 ± 10.0	< 0.001
RVEDA, cm^2^	25.3 ± 7.2	18.0 (7.8)	< 0.001
RVESA, cm^2^	18.2 ± 6.8	11.0 (6.3)	< 0.001
RV basal diameter, mm	45.9 ± 6.9	38.0 (9.0)	< 0.001
D_MPA_, mm	32.0 (6.2)	30.0 (5.5)	0.034
D_RPA_, mm	24.0 (4.4)	23.5 (4.0)	0.180
D_LPA_, mm	22.0 (4.0)	20.0 (3.3)	0.001
LVEF, %	65.2 ± 5.1	65.3 ± 4.8	0.961
Strain parameters			
STE-RVFWLS, %	15.7 (7.6)	21.0 (10.8)	< 0.001
STE-RVGLS, %	13.4 ± 4.4	17.1 ± 5.1	< 0.001
Hemodynamics			
CVP, mmHg	8.1 ± 4.1	6.0 (4.0)	0.019
mPAP, mmHg	50.8 ± 10.4	35.5 ± 11.9	< 0.001
PCWP, mmHg	9.0 (5.5)	9.5 ± 3.3	0.780
PVR, dyn·s·cm^−5^	888.7 ± 363.5	545.4 ± 383.8	< 0.001
CO, L/min	3.9 (1.4)	4.0 (1.3)	0.103
CI, L/min/m^2^	2.3 ± 0.5	2.5 ± 0.6	0.014
Clinical indicators			
WHO FC			
I/II/III/IV	4/40/29/3	19/47/9/1	< 0.001
6WMD, m	385.5 (126.0)	454.5 (85.5)	< 0.001
NT-proBNP, ng/L	675.0 (1858.0)	134.0 (276.0)	< 0.001

Values are expressed as mean ± SD, median (IQR), or frequency (percentage), as appropriate

*Abbreviations*: *PASP* Pulmonary artery systolic pressure by echocardiography, *TAPSE* Tricuspid annular plane systolic excursion, *LVEI* Left ventricular eccentric index, *TRV* Tricuspid regurgitation peak velocity, *D*_*IVC*_ Diameter of inferior vena cava, *S'* Peak systolic velocity of the tricuspid annulus, *RIMP* Right ventricular index of myocardial performance, *FAC* Right ventricular fractional area change, *RVEDA* RV end-diastolic area, *RVESA* RV end-systolic area; D_MPA_, main pulmonary artery diameter, *D*_*RPA*_ Right pulmonary artery diameter, *D*_*LPA*_ Left pulmonary artery diameter, *LVEF* Left ventricle ejection fraction, *STE-RVFWLS* Speckle tracking echocardiography-RV free wall longitudinal strain, *STE-RVGLS* Speckle tracking echocardiography-RV global longitudinal strain, *CVP* Central venous pressure, *mPAP* Mean pulmonary artery pressure, *PVR* Pulmonary vascular resistance, *PCWP* Pulmonary capillary wedge pressure, *CO* Cardiac output, *CI* Cardiac index, *WHO FC* World Health Organization Functional Class, *6MWD* 6 min walking distance, *NT-proBNP* N-terminal probrain natriuretic peptide

### Predictors of BPA Outcomes

**Fig. 4:**
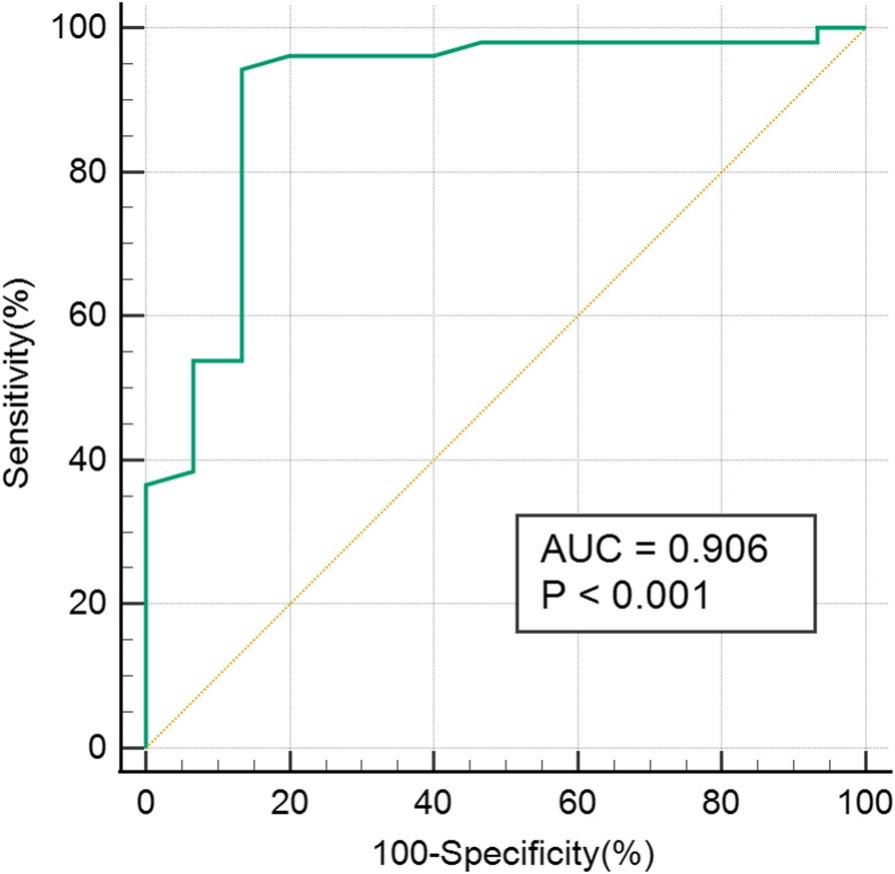
Receiver operating characteristic curve analysis demonstrating the ability of right ventricular free wall longitudinal strain (RVFWLS) to predict BPA responders. AUC, area under the curve

**Fig. 5:**
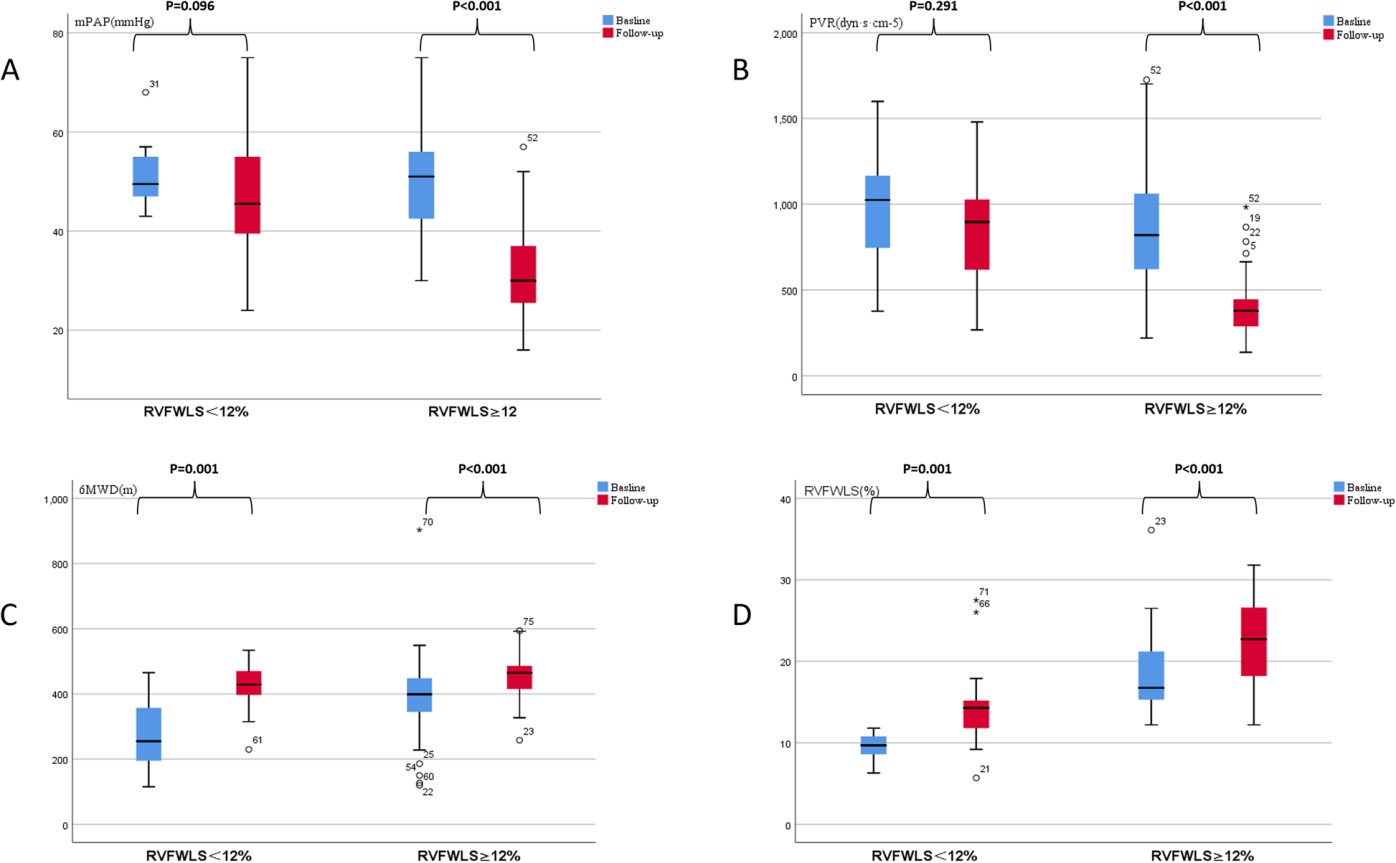
Hemodynamics, 6-min walking distance (6MWD) and right ventricular free wall longitudinal strain (RVFWLS) at baseline and follow-up in chronic thromboembolic pulmonary hypertension (CTEPH) patients with RVFWLS ≥ 12% and < 12% mPAP (**A**), PVR (**B**), 6MWD (**C**), and RVFWLS(D) at baseline and follow-up in CTEPH patients with RVFWLS ≥ 12% and < 12%. At baseline, RVFWLS < 12% vs RVFWLS ≥ 12% (mPAP: 51.4 ± 6.4 mmHg vs 50.3 ± 10.4 mmHg, *p* = 0.669; PVR: 1008.5 ± 326.1 dyn·s·cm-5 vs 845.8 ± 333.8 dyn·s·cm-5,* p* = 0.088; 6MWD: 275.3 ± 110.4 m vs 394.7 ± 121.4 m, *p* = 0.001; RVFWLS: 9.5 ± 1.6% vs 18.2 ± 4.3%,* p* < 0.001). mPAP, mean pulmonary artery pressure; PVR, pulmonary vascular resistance

The results of regression analysis are presented in Table 4. In the univariable logistic regression, TAPSE, EI RVEDA, RVESA, STE-RVFWLS, STE-RVGLS, WHO FC, and 6MWD were associated with *p* values < 0.05. RVEDA and RVESA were excluded from multivariable logistic regression owing to their collinearity with FAC. Finally, six variables were included in the multivariate regression model. Of these, only RVFWLS was identified as an independent predictor of BPA outcomes (odds ratio [OR]: 2.28, 95% confidence interval [CI] 1.147–4.532, *p* = 0.019). The strongest predictors in the multivariable analysis were included in the ROC curve analysis. Based on the largest Youden index, the optimal RVFWLS cut-off value for predicting BPA responders was 12% (area under the curve [AUC]: 0.906 [95% CI 0.807–1.000]) (Fig. 4). We further subclassified patients based on whether RVFWLS was ≥ 12% or < 12% (Fig. 5). At baseline, patients with RVFWLS < 12% and RVFWLS ≥ 12% showed a significant difference in 6MWD and RVFWLS, but were comparable in terms of mPAP and PVR (6MWD: 275.3 ± 110.4 m vs 394.7 ± 121.4 m, *p* = 0.001; RVFWLS: 9.5 ± 1.6% vs 18.2 ± 4.3%, *p* < 0.001; mPAP: 51.4 ± 6.4 mmHg vs 50.3 ± 10.4 mmHg, *p* = 0.669; PVR: 1008.5 ± 326.1 dyn·s·cm-5 vs 845.8 ± 333.8 dyn·s·cm-5, *p* = 0.088). The results showed a larger change in mPAP (*p* < 0.001), PVR (*p* < 0.001), 6MWD (*p* < 0.001), and RVFWLS (*p* < 0.001) in patients with RVFWLS ≥ 12% at baseline. 6MWD (*p* = 0.001) and RVFWLS (*p* = 0.001) also showed notable improvement after BPA in CTEPH patients with RVFWLS < 12%. However, there was no significant decrease in mPAP (*p* = 0.096) and PVR (*p* = 0.291) in CTEPH patients who had RVFWLS < 12% at baseline.

**Table 4:** Univariable and multivariable logistic regression analysis for BPA responders

Univariable logistic regression				Multivariable logistic regression		
Variable	**OR**	**95% CI**	***P* value**	**OR**	**95% CI**	***P* value**
Age, years	1.021	0.979–1.065	0.338			
Female, n (%)	1.137	0.387–3.336	0.815			
BSA (m^2^)	0.254	0.012–5.172	0.373			
PASP, mmHg	0.975	0.948–1.002	0.072			
TAPSE, mm	1.218	1.013–1.463	0.035	0.852	0.563–1.289	0.449
LVEI	0.037	0.003–0.513	0.014	0.042	0.001–3.052	0.147
TRV, cm/s	0.990	0.980–1.000	0.052			
D_IVC_, mm	0.923	0.825–1.033	0.165			
S', cm/s	0.953	0.765–1.186	0.666			
RIMP	1.171	0.022–1.328	0.091			
FAC, %	1.059	0.999–1.123	0.056			
RVEDA, cm^2^	0.896	0.824–0.973	0.010			
RVESA, cm^2^	0.891	0.816–0.972	0.009			
RV basal diameter, mm	0.927	0.857–1.003	0.059			
D_MPA,_ mm	0.966	0.894–1.043	0.376			
D_RPA,_ mm	0.965	0.859–1.084	0.546			
D_LPA,_ mm	0.986	0.878–1.108	0.812			
LVEF, %	0.961	0.868–1.065	0.451			
STE-RVFWLS, %	1.768	1.320–2.368	< 0.001	2.280	1.147–4.532	0.019
STE-RVGLS, %	1.736	1.277–2.361	< 0.001	0.751	0.361–1.559	0.442
CVP, mmHg	0.930	0.820–1.055	0.262			
mPAP, mmHg	0.980.0.938	0.936–1.033	0.507			
PCWP, mmHg	1.000	0.842–1.045	0.244			
PVR, dyn·s·cm^−5^	1.119	0.998–1.001	0.712			
CO, L/min	1.378	0.703–1.783	0.635			
CI, L/min/m^2^		0.472–4.021	0.558			
WHO FC						
I/II/III/IV	0.362	0.154–0.853	0.020	2.541	0.416–15.504	0.321
6WMD, mm	1.006	1.001–1.012	0.013	1.004	0.995–1.102	0.403
NT-proBNP, ng/L	1.000	1.000–1.000	0.131			
Number of BPA sessions	1.114	0.837–1.482	0.460			
Time Internal, days	1.000	0.999–1.001	0.881			

*Abbreviations*: *BSA* Body surface area, *PASP* Pulmonary artery systolic pressure by echocardiography, *TAPSE* Tricuspid annular plane systolic excursion, *LVEI* Left ventricular eccentric index, *TRV* Tricuspid regurgitation peak velocity, *D*_*IVC*_ Diameter of inferior vena cava, *S'* Peak systolic velocity of the tricuspid annulus, *RIMP* Right ventricular index of myocardial performance, *FAC* Right ventricular fractional area change, *RVEDA* RV end-diastolic area, *RVESA* RV end-systolic area, *D*_*MPA*_ Main pulmonary artery diameter; D_RPA_, right pulmonary artery diameter, *D*_*LPA*_ Left pulmonary artery diameter, *LVEF* Left ventricle ejection fraction, *STE-RVFWLS* Speckle tracking echocardiography-RV free wall longitudinal strain, *STE-RVGLS* Speckle tracking echocardiography-RV global longitudinal strain, *CVP* Central venous pressure, *mPAP* Mean pulmonary artery pressure, *PVR* Pulmonary vascular resistance, *PCWP* Pulmonary capillary wedge pressure, *CO* Cardiac output, *CI* Cardiac index, *WHO FC* World Health Organization Functional Class, *6MWD* 6 min walking distance, *NT-proBNP* N-terminal probrain natriuretic peptide, *BPA* Balloon pulmonary angioplasty

### Intra-observer and inter-observer agreement regarding 2D-STE

**Fig. 6:**
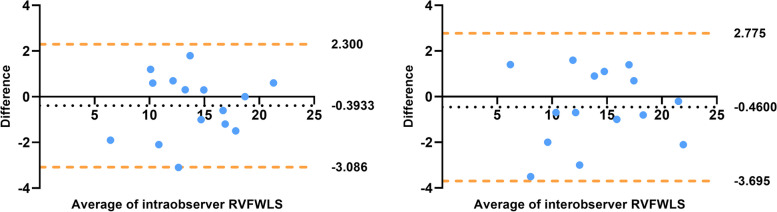
Bland–Altman plots for intra-observer and inter-observer agreement of 2D-STE findings. The horizontal dots represent the mean difference, and the yellow lines represent the mean difference ± 1.96 standard deviations

Bland–Altman plots showed good intra-observer (bias: -0.3933, 95% limits of agreement [LoA] -3.086–2.300) and inter-observer (bias: -0.4600, 95% LoA -3.695–2.775) agreement. Almost all the points were within the 95% LoA (Table 5, Fig. 6).

**Table 5:** Intra-observer and inter-observer reproducibility

	**Bias (SD)**	**95% Limits of Agreement**
Intra-observer analysis		
RVFWLS	-0.39 (1.374)	-3.086 to 2.300
Inter-observer analysis		
RVFWLS	-0.46 (1.650)	-3.695 to 2.775

*Abbreviations*: *SD* Standard deviation, *RVFWLS* RV free wall longitudinal strain, *RVGLS* RV global longitudinal strain

## Discussion

The key findings of this study are as follows: (1) BPA seems to be effective in improving hemodynamics and inducing RV reverse remodeling. (2) RV remodeling plays an important role in the efficacy of BPA. RVFWLS ≥ 12% at baseline was found to be an independent predictor of the outcomes of BPA.

CTEPH is a potentially life-threatening condition secondary to acute pulmonary embolism and is treatable [4, 17]. The response of the RV to increased afterload is a key determinant of morbidity and mortality in patients with PH [18]. RV adaptive fibrosis plays an important role in the early stages of the development of PH. This adaptation is characterized by concentric hypertrophy of the RV myocardium with almost no change in chamber volume. In later stages, maladaptive fibrosis increases myocardial stiffness, disturbs cardiomyocyte excitation–contraction coupling, and perturbs the cardiac contraction coordination [6, 7]. Studies have shown that RV fibrosis is closely associated with RV function and is generally considered to have a negative impact on function [19]. Chronically increased afterload leads to the maladaptive remodeling of the RV, resulting in progressive right heart failure [7, 18]. RVFWLS obtained by 2D-STE has been shown to be a sensitive marker of the changes reflecting the remodeling in RV function [2, 20]. Echocardiography can enable prompt structural and functional assessment of the right-sided heart and the hemodynamics [4]. The ability to predict the efficacy of BPA in CTEPH by echocardiography can help avoid unnecessary invasive RHC to some extent.

### Efficacy of BPA

The 2022 ESC/ERS guidelines for PH recommend a multimodal therapeutic strategy for CTEPH including pharmacotherapy, BPA, and PEA. In previous studies, PH drugs seemed to have a small beneficial effect on prognosis. Riociguat, a PH targeted-drug, can improve hemodynamics and exercise performance. However, the effects of the drugs in reducing mPAP and PVR are not as remarkable as BPA [21]. For CTEPH patients with technically accessible pulmonary arterial obstructions, PEA is the primary treatment that may normalize pulmonary hemodynamics [4, 22]. PEA is a potential cure for CTEPH patients, but only a limited number of cases are PEA candidates due to inaccessible lesion sites [23–25]. No more than 60% of patients undergo PEA [24]. Patients with CTEPH in whom PEA is not indicated have a poor prognosis after surgery [26]. The CTEPH patients included in this study had not undergone PEA for technical reasons. Historically, BPA has not been widely used in many centers due to previously reported high complication rates [27]. At present, BPA can be safely performed in expert centers with acceptable rates of complications and mortality [27]. BPA is suitable for patients who are not candidates for PEA due to distal-type CTEPH or severe concomitant comorbidity, and for patients with residual PH after PEA. A multicenter study demonstrated the efficacy of BPA in significantly improving hemodynamic, functional, and biochemical parameters [16], which is consistent with our findings. The present study suggests that BPA can offer distinct benefits in the treatment of patients with CTEPH.

Due to the complexity of the right ventricular anatomy, RV function assessment is challenging [28]. A previous study demonstrated that echocardiography provides a comprehensive assessment of RV reverse remodeling (including structure and function) in CTEPH patients undergoing BPA [29]. The present study showed consistent findings in a larger sample of patients in addition to confirming a significant improvement in PASP after BPA. However, some patients in our study still had poor RV function and hemodynamics after BPA.

### Baseline RVFWLS predicts the outcomes of BPA

The treatment goals for CTEPH patients have not been clarified, and the current standard still relies on hemodynamic parameters [4]. In the present study, patients with mPAP ≤ 30 mmHg or those showing PVR decrease by ≥ 30% were considered as responders. At present, there is no widely accepted method to predict the efficacy of BPA [30]. Most experts accept that RV function is a major determinant of prognosis in patients with CTEPH [1]. Similarly, in univariable logistic regression analysis, functional markers, rather than hemodynamic parameters, showed the strongest association with BPA outcomes. In multivariable logistic regression analysis, we observed a stronger correlation between RVFWLS at baseline and outcomes of BPA. RVFWLS is a reliable marker of right ventricular systolic function. In our study, responders had a higher RVFWLS than non-responders, even though the hemodynamic parameters were comparable at baseline. This indicated that the level of RVFWLS at baseline influenced the outcomes of BPA and RVFWLS ≥ 12% was the best predictor of BPA responders. Our previous study with a small sample size also yielded consistent findings: baseline RVFWLS > 12.2% was found to predict outcomes of BPA [31].

In a previous study, the diameter of the main pulmonary artery obtained by CT was found to predict the therapeutic effect of BPA in patients with CTEPH [32]. We did not observe this phenomenon in our study. This may be attributed to the use of different assessment techniques and different effect variables. WHO FC and 6MWD both showed significance in the univariable logistic regression analysis. The fact that these are not highly objective measures explains the lack of significant association observed in multivariate regression analysis [32]. TAPSE has its limitations as it only partially reflects the RV function [20]. RVGLS is affected by left ventricular function because of the common interventricular septum. An interesting observation was the lack of correlation between baseline hemodynamic parameters and post-BPA outcomes, which is similar to a previous study that used the same criteria to define BPA results [12]. Further studies are required to understand the mechanism.

Despite the high diagnostic value of RHC for PH, it is not suitable for frequent use due to its invasive nature and high cost. Therefore, a readily available technique for routine monitoring of the prognosis of patients with CTEPH can provide distinct leverage in clinical settings. RVFWLS obtained by 2D-STE has been used to assess RV function; therefore, strain echocardiography can be used to predict the outcomes of BPA in patients with CTEPH to a certain extent [11]. BPA has a promising effect in CTEPH patients, but there is currently no established consensus on the indications for BPA [12]. The conclusions from this study can help optimize clinical strategies for screening candidates for BPA because patients with RVFWLS ≥ 12% were found to be suitable candidates for BPA. We do not consider that RVFWLS < 12% is a contraindication for BPA, but rather that an individualized treatment plan should be formed for these patients after careful evaluation.

### Limitations

Some limitations of this study should be considered. First, this was a single-center, small-sample retrospective study. Therefore, larger prospective studies are required to confirm our findings. Second, most of the patients in this study did not undergo RHC and echocardiography on the same day, which may have resulted in a poor correlation between the two. Lastly, we did not examine the effects of medical therapy on the efficacy of BPA. This was because most patients were prescribed a combination of drugs, and the timing and dosage of the drugs were unclear. In a previous study, PH-targeted drugs were not found to predict BPA response or non-response [33].

## Conclusion

BPA can effectively reduce pulmonary arterial pressure, reverse right ventricular remodeling, and improve exercise capacity. RVFWLS may predict the outcomes of BPA and provide a reference for clinical screening of patients before BPA treatment.

## Data Availability

All data used and analyzed during this study are included in the manuscript.

## Abbreviations

2D-STE: 2D-speckle tracking echocardiography
6MWD: 6 Minutes walking distance
ASE: American Society of Echocardiography
AUC: Area under the curve
BPA: Balloon pulmonary angioplasty
BSA: Body surface area
CI: Cardiac index
CO: Cardiac output
CTEPH: Chronic thromboembolic pulmonary hypertension
CVP: Central venous pressure
D_IVC_: Diameter of inferior vena cava
D_LPA_: Left pulmonary artery diameter
D_MPA_: Main pulmonary artery diameter
D_RPA_: Right pulmonary artery diameter
ERS: European Respiratory Society
ESC: European Society of Cardiology
FAC: Right ventricular fractional area change
LoA: Limits of Agreement
LVEF: Left ventricle ejection fraction
LVEI: Left ventricular eccentric index
mPAP: Mean pulmonary artery pressure
NT-proBNP: N-terminal probrain natriuretic peptide
PASP: Pulmonary artery systolic pressure by echocardiography
PCWP: Pulmonary capillary wedge pressure
PEA: Pulmonary endarterectomy
PH: Pulmonary hypertension
PVR: Pulmonary vascular resistance
RHC: Right cardiac catheterization
RIMP: Right ventricular index of myocardial performance
ROC: Receiver operating characteristic
RV: Right ventricular
RVEDA: RV end-diastolic area
RVESA: RV end-systolic area
S': Peak systolic velocity of the tricuspid annulus
STE-RVFWLS: Speckle tracking echocardiography-RV free wall longitudinal strain
STE-RVGLS: Speckle tracking echocardiography-RV global longitudinal strain
TAPSE: Tricuspid annular plane systolic excursion;
TRV: Tricuspid regurgitation peak velocity
WHO FC: World Health Organization Functional Class

## References

[1] Li W, Yang T, Quan RL, Chen XX, An J, Zhao ZH, Liu ZH, Xiong CM, He JG, Gu Q (2021). Balloon pulmonary angioplasty reverse right ventricular remodelling and dysfunction in patients with inoperable chronic thromboembolic pulmonary hypertension: a systematic review and meta-analysis. Eur Radiol.

[2] Yuchi Y, Suzuki R, Kanno H, Teshima T, Matsumoto H, Koyama H (2021). Right Ventricular Myocardial Adaptation Assessed by Two-Dimensional Speckle Tracking Echocardiography in Canine Models of Chronic Pulmonary Hypertension. Front Vet Sci.

[3] Fukui S, Ogo T, Morita Y, Tsuji A, Tateishi E, Ozaki K, Sanda Y, Fukuda T, Yasuda S, Ogawa H (2014). Right ventricular reverse remodelling after balloon pulmonary angioplasty. Eur Respir J.

[4] Humbert M, Kovacs G, Hoeper MM, Badagliacca R, Berger RMF, Brida M, Carlsen J, Coats AJS, Escribano-Subias P, Ferrari P (2022). 2022 ESC/ERS Guidelines for the diagnosis and treatment of pulmonary hypertension. Eur Heart J.

[5] Otani N, Watanabe R, Tomoe T, Toyoda S, Yasu T, Nakamoto T (2023). Pathophysiology and treatment of chronic thromboembolic pulmonary hypertension. Int J Mol Sci.

[6] Llucià-Valldeperas A, de Man FS, Bogaard HJ (2021). Adaptation and Maladaptation of the Right Ventricle in Pulmonary Vascular Diseases. Clin Chest Med.

[7] Rako ZA, Kremer N, Yogeswaran A, Richter MJ, Tello K (2023). Adaptive versus maladaptive right ventricular remodelling. ESC Heart Fail.

[8] Chen BX, Xing HQ, Gong JN, Guo XJ, Xi XY, Yang YH, Huo L, Yang MF (2022). Imaging of cardiac fibroblast activation in patients with chronic thromboembolic pulmonary hypertension. Eur J Nucl Med Mol Imaging.

[9] Sumimoto K, Tanaka H, Mukai J, Yamashita K, Tanaka Y, Shono A, Suzuki M, Yokota S, Suto M, Takada H (2020). Effects of balloon pulmonary angioplasty for chronic thromboembolic pulmonary hypertension on remodeling in right-sided heart. Int J Cardiovasc Imaging.

[10] Ostenfeld E, Kjellström B (2020). The Conundrum of Right Ventricular Remodeling and Outcome in Pulmonary Hypertension. Circ Cardiovasc Imaging.

[11] Park JH, Kusunose K, Kwon DH, Park MM, Erzurum SC, Thomas JD, Grimm RA, Griffin BP, Marwick TH, Popovic ZB (2015). Relationship between right ventricular longitudinal strain, invasive hemodynamics, and functional assessment in pulmonary arterial hypertension. Korean Circ J.

[12] Taniguchi Y, Brenot P, Jais X, Garcia C, Weatherald J, Planche O, Fadel E, Humbert M, Simonneau G (2018). Poor Subpleural perfusion predicts failure after balloon pulmonary angioplasty for Nonoperable chronic thromboembolic pulmonary hypertension. Chest.

[13] Mitchell C, Rahko PS, Blauwet LA, Canaday B, Finstuen JA, Foster MC, Horton K, Ogunyankin KO, Palma RA, Velazquez EJ (2019). Guidelines for performing a comprehensive transthoracic echocardiographic examination in adults: recommendations from the American society of echocardiography. J Am Soc Echocardiogr.

[14] Gonzalez-Hermosillo LM, Cueto-Robledo G, Roldan-Valadez E, Graniel-Palafox LE, Garcia-Cesar M, Torres-Rojas MB, Romero-Martinez B, Castro-Escalante KY (2022). Right Heart Catheterization (RHC): a comprehensive review of provocation tests and hepatic Hemodynamics in patients with Pulmonary Hypertension (PH). Curr Probl Cardiol.

[15] Olsson KM, Wiedenroth CB, Kamp JC, Breithecker A, Fuge J, Krombach GA, Haas M, Hamm C, Kramm T, Guth S (2017). Balloon pulmonary angioplasty for inoperable patients with chronic thromboembolic pulmonary hypertension: the initial German experience. Eur Respir J.

[16] Darocha S, Roik M, Kopec G, Araszkiewicz A, Furdal M, Lewandowski M, Jachec W, Grabka M, Banaszkiewicz M, Pietrasik A (2022). Balloon pulmonary angioplasty in chronic thromboembolic pulmonary hypertension: a multicentre registry. EuroIntervention.

[17] Valerio L, Mavromanoli AC, Barco S, Abele C, Becker D, Bruch L, Ewert R, Faehling M, Fistera D, Gerhardt F (2022). Chronic thromboembolic pulmonary hypertension and impairment after pulmonary embolism: the FOCUS study. Eur Heart J.

[18] Mendiola EA, da Silva GoncalvesBos D, Leichter DM, Vang A, Zhang P, Leary OP, Gilbert RJ, Avazmohammadi R, Choudhary G (2023). Right ventricular architectural remodeling and functional adaptation in pulmonary hypertension. Circ Heart Fail.

[19] Bekedam FT, Goumans MJ, Bogaard HJ, de Man FS, Llucia-Valldeperas A (2023). Molecular mechanisms and targets of right ventricular fibrosis in pulmonary hypertension. Pharmacol Ther.

[20] Carluccio E, Biagioli P, Lauciello R, Zuchi C, Mengoni A, Bardelli G, Alunni G, Gronda EG, Ambrosio G (2019). Superior prognostic value of right ventricular free wall compared to global longitudinal strain in patients with heart failure. J Am Soc Echocardiogr.

[21] Jais X, Brenot P, Bouvaist H, Jevnikar M, Canuet M, Chabanne C, Chaouat A, Cottin V, De Groote P, Favrolt N (2022). Balloon pulmonary angioplasty versus riociguat for the treatment of inoperable chronic thromboembolic pulmonary hypertension (RACE): a multicentre, phase 3, open-label, randomised controlled trial and ancillary follow-up study. Lancet Respir Med.

[22] Guth S, Mayer E, Prüfer D, Wiedenroth CB (2022). Pulmonary endarterectomy: technique and pitfalls. Annals of Cardiothoracic Surgery.

[23] Ghofrani HA, Simonneau G, D'Armini AM, Fedullo P, Howard LS, Jais X, Jenkins DP, Jing ZC, Madani MM, Martin N (2017). Macitentan for the treatment of inoperable chronic thromboembolic pulmonary hypertension (MERIT-1): results from the multicentre, phase 2, randomised, double-blind, placebo-controlled study. Lancet Respir Med.

[24] Martin-Suarez S, Loforte A, Cavalli GG, Gliozzi G, Botta L, Mariani C, Orioli V, Votano D, Costantino A, Santamaria V (2022). Therapeutic alternatives in chronic thromboembolic pulmonary hypertension: from pulmonary endarterectomy to balloon pulmonary angioplasty to medical therapy. State of the art from a multidisciplinary team. Ann Cardiothorac Surg.

[25] Ghofrani HA, Kim NH (2022). Medical and interventional therapies for inoperable CTEPH: a necessary combination?. Lancet Respir Med.

[26] Borchers A, Pieler T (2010). Programming pluripotent precursor cells derived from Xenopus embryos to generate specific tissues and organs. Genes (Basel).

[27] Wiedenroth CB, Deissner H, Adameit MSD, Kriechbaum SD, Ghofrani HA, Breithecker A, Haas M, Roller F, Rolf A, Hamm CW (2022). Complications of balloon pulmonary angioplasty for inoperable chronic thromboembolic pulmonary hypertension: Impact on the outcome. J Heart Lung Transplant.

[28] Lang RM, Badano LP, Mor-Avi V, Afilalo J, Armstrong A, Ernande L, Flachskampf FA, Foster E, Goldstein SA, Kuznetsova T (2015). Recommendations for cardiac chamber quantification by echocardiography in adults: an update from the American Society of Echocardiography and the European Association of Cardiovascular Imaging. Eur Heart J Cardiovasc Imaging.

[29] Broch K, Murbraech K, Ragnarsson A, Gude E, Andersen R, Fiane AE, Andreassen J, Aakhus S, Andreassen AK (2016). Echocardiographic evidence of right ventricular functional improvement after balloon pulmonary angioplasty in chronic thromboembolic pulmonary hypertension. J Heart Lung Transplant.

[30] Zhang Y, Li X, Luo Q, Zhao Q, Zeng Q, Yang T, Jin Q, Yan L, Duan A, Ma X (2022). Heart-rate recovery at 1 min after exercise predicts response to balloon pulmonary angioplasty in patients with inoperable chronic thromboembolic pulmonary hypertension. Front Cardiovasc Med.

[31] Zhang X, Guo D, Wang J, Gong J, Wu X, Jiang Z, Zhong J, Lu X, Yang Y, Li Y (2020). Speckle tracking for predicting outcomes of balloon pulmonary angioplasty in patients with chronic thromboembolic pulmonary hypertension. Echocardiography.

[32] Tsukada J, Yamada Y, Kawakami T, Matsumoto S, Inoue M, Nakatsuka S, Okada M, Fukuda K, Jinzaki M (2021). Treatment effect prediction using CT after balloon pulmonary angioplasty in chronic thromboembolic pulmonary hypertension. Eur Radiol.

[33] de Perrot M, Donahoe L, McRae K, Thenganatt J, Moric J, Chan J, McInnis M, Jumaa K, Tan KT, Mafeld S (2022). Outcome after pulmonary endarterectomy for segmental chronic thromboembolic pulmonary hypertension. J Thorac Cardiovasc Surg.

